# Arbuscular Mycorrhizal Fungi Trigger Transcriptional Expression of Flavonoid and Chlorogenic Acid Biosynthetic Pathways Genes in Tomato against *Tomato Mosaic Virus*

**DOI:** 10.1038/s41598-019-46281-x

**Published:** 2019-07-04

**Authors:** Dalia G. Aseel, Younes M. Rashad, Saad M. Hammad

**Affiliations:** Plant Protection and Biomolecular Diagnosis Department, Arid Lands Cultivation Research Institute, City of Scientific Research and Technological Applications, New Borg El-Arab City, 21934 Egypt

**Keywords:** Arbuscular mycorrhiza, Biotic

## Abstract

Tomato mosaic disease, caused by *Tomato Mosaic Virus* (ToMV), is one of the most destructive diseases which results in serious crop losses. Research investigations dealing with the biocontrol activity of arbuscular mycorrhizal fungi (AMF) against this viral disease are limited. In this study, the biocontrol activity of AMF on tomato plants infected with ToMV was evaluated in the greenhouse. In addition, their impacts on the transcriptional expression levels of thirteen genes controlling the phenylpropanoid, flavonoid and chlorogenic acid biosynthetic pathways were also investigated using quantitative real-time PCR. Transcriptional expressions of the majority of the studied genes were up-regulated by mycorrhizal colonization in the presence of ToMV, particularly *PAL1* and *HQT*, suggesting their pathogen-dependent inducing effect. Under greenhouse conditions, a significant reduction in the disease severity and incidence, as well as the viral accumulation level was observed as a response to the mycorrhizal colonization of the infected plants. Moreover, the evaluated growth parameters, photosynthetic pigments, and flavonoid content were significantly enhanced by AMF colonization. The obtained results demonstrated the protective role of AMF in triggering the plant immunity against ToMV in a pathogen-dependent manner. Beside their protective and growth-promotion activities, AMF are characterized by low-cost and environment-friendly properties which support their possible use for control of tomato mosaic disease.

## Introduction

Tomato (*Solanum lycopersicum* L.) is one of the most economic crops in the world. In Egypt, it represents a highly strategic vegetable crop. Indeed, Egypt is the fifth major tomato-producing country in the world. The area under tomato cultivation in 2017 was 182,444 ha with a total production of 7,297,100 tons^1^. However, tomato crop suffers from attack by various pathogens including fungi, bacteria, and viruses^2,3^.

Tomato mosaic disease, caused by *Tomato Mosaic Virus* (ToMV), is one of the most destructive diseases which results in serious losses affecting size, quality, and production of the tomato fruits worldwide^4^. ToMV (a member of the *Tobamovirus* genus) has a wide host range and is transmitted primarily as seed-borne or by mechanical contact with contaminated growing tools, farmer’s activities, or infected plants^5^. Due to its ability to survive in the dried plant debris for up to 50 years, control of ToMV is difficult. Their management depends mainly on excluding the infected plants, use of certified virus-free seeds or seedlings, and selection of ToMV-resistant plant cultivars^6^. One of the promising safe approaches for the management of viral plant diseases is the use of biocontrol agents which have the potentiality to reduce the disease occurrence and promote the plant growth. Various biocontrol agents have been reported in this regard such as *Trichoderma* spp., *Pseudomonas* spp., *Bacillus* spp., and *Streptomyces* spp.^7–9^.

Arbuscular mycorrhizal fungi (AMF) are a category of obligate biotrophs (phylum. Mucoromycota, subphylum. Glomeromycotina)^10^ that form a mutualistic symbiotic association with roots of most terrestrial plants. AMF play vital roles in improving the plant growth, water and nutrients uptake, plant tolerance to salinity and drought, and resistance to plant diseases^11,12^. Induction of the plant immune system using AMF against various fungal^13^, bacterial^14^, and nematode diseases^15^ has been reported by many researchers during the last years, while, there are limited studies on the biocontrol activity of AMF against viral plant diseases. In this regard, Maffei *et al*.^16^ reported a significant reduction in the disease symptoms of tomato plants infected with tomato yellow leaf curl Sardinia virus, as well as the viral DNA concentration when colonized with AMF. One of the defense-related responses triggered in the plant by AMF colonization is the accumulation of antimicrobial phenolic compounds^13^. Flavonoids and chlorogenic acid are polyphenolic compounds which have various functions in the plant including resistance against UV radiation, heat, pathogenic agents (fungi, bacteria and viruses), and herbivores, in addition to their antioxidant role^17,18^. Their antiviral properties have been reported not only against the plant viruses but also against viruses that infect human^19,20^. Their antiviral mechanisms include binding to the viral DNA and/or capsid proteins, and inhibition of the viral polymerase and integrase enzymes^21^. The present study was planned to evaluate the biocontrol potential of AMF against ToMV on tomato plants as well as their effects on the transcriptional expression levels of flavonoid and chlorogenic acid biosynthetic pathways genes.

## Results

### Disease assessment

**Figure 1:**
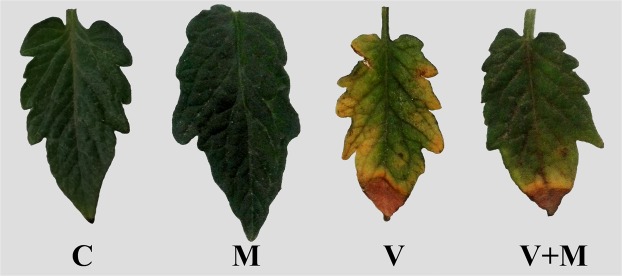
A photograph showing the disease symptoms on tomato leaves infected with ToMV in response to mycorrhizal colonization, where, C: untreated control, M: colonized with AMF, V: infected with ToMV, and V + M: infected with ToMV and colonized with AMF.

Typical symptoms of tomato mosaic virus, including mosaic, mottling, yellowing, necrosis, malformation and size reduction of the plant leaves were observed on tomato plants infected with ToMV (Fig. 1). Data presented in (Table 1) show the disease severity and incidence in response to the applied treatments. Mycorrhizal colonization of ToMV-infected plants significantly reduced both disease severity and incidence compared with the non mycorrhizal-ToMV-infected plants. No disease symptoms were observed on the uninfected plants.

**Table 1:** Disease assessment of tomato plants infected with ToMV (21 days after inoculation) in response to root mycorrhizal colonization.

Treatment*	Disease incidence (%)	Disease incidence grade	PDI (%)
C	0^c^	—	0^c^
M	0^c^	—	0^c^
V	86.55^a^	High	80.67^a^
V + M	37.55^b^	moderate	26.34^b^

C = untreated control, M = colonized with AMF, V = infected with ToMV, and PDI = percent disease index.

*Values of each column followed by the same letter are not significantly different according to Duncan’s multiple range test (*P* ≤ 0.05), each value represents the mean of four replicates.

### Mycorrhizal colonization assessment

**Figure 2:**
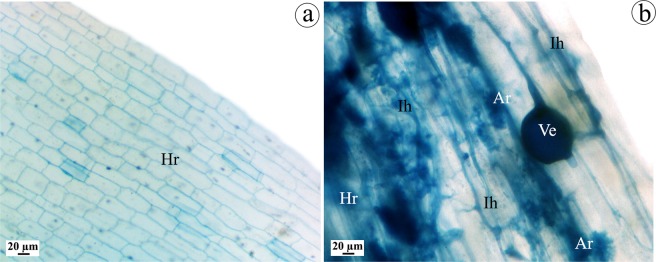
Tomato root showing typical mycorrhizal colonization structures. Non-treated control root (**a**), and AMF-colonized root (**b**), where, Hr = host root, Ih = interaradical hyphae, Ve = vesicle, and Ar = arbuscule.

Levels of the mycorrhizal colonization in tomato roots of the applied treatments are presented in (Table 2). Roots of the uninfected-AMF-treated tomato plants showed high levels of the evaluated colonization parameters (colonization frequency and intensity, and arbuscules frequency), recording 88.33, 56.33, and 26.67%, respectively. Typical mycorrhizal colonization structures were observed in roots of AMF-treated tomato plants (Fig. 2). Colonization levels of the infected-AMF-treated plants showed non-significant reduction compared to the uninfected-AMF-treated plants. No mycorrhizal colonization was observed in the non-AMF-treated tomato plants.

**Table 2:** Colonization level of AMF in the roots of tomato plants infected with ToMV (21 days after inoculation).

Treatment*	F (%)	MI (%)	A (%)
C	0^b^	0^c^	0^c^
M	88.33 ± 4.6^a^	56.33 ± 3.7^a^	26.67 ± 2.4^a^
V	0^b^	0^c^	0^c^
V + M	87.55 ± 3.1^a^	53.66 ± 4.2^a^	25.60 ± 2.1^a^

C = untreated control, M = colonized with AMF, and V = infected with ToMV, F = frequency of root colonization, MI = intensity of cortical colonization, and A = arbuscules frequency.

*Values of each column followed by the same letter are not significantly different according to Duncan’s multiple range test (*P* ≤ 0.05), each value represents the mean of four replicates ± SD.

### Effects on the growth parameters

The obtained results showed that infection with ToMV significantly reduced the lengths and dry weights of tomato shoot and roots compared with the control plants, while, the number of leaves did not show any significant difference (Table 3). However, root colonization with AMF significantly enhanced all evaluated growth parameters recording the highest values when compared to the other treatments. Except for the number of leaves, AMF-colonization of the ToMV-infected plants significantly reduced the negative effects, resulted from the viral infection, on the tested growth parameters compared to the non-mycorrhizal ToMV-infected plants.

**Table 3:** Effect of root colonization with AMF on the growth parameters of tomato plants infected with ToMV (21 days after inoculation).

Treatment*	Shoot length (cm)	Root length (cm)	Shoot dry weight (g)	Root dry weight (g)	No. of leaves
C	22.9 ± 0.33^b^	27.0 ± 0.57^b^	1.23 ± 0.03^b^	0.27 ± 0.02^b^	7.66 ± 0.33^b^
M	26.7 ± 0.33^a^	33.3 ± 0.25^a^	1.93 ± 0.12^a^	0.36 ± 0.06^a^	9.33 ± 0.33^a^
V	18.2 ± 0.83 ^c^	22.0 ± 0.55^c^	0.98 ± 0.02^c^	0.20 ± 0.05^c^	7.00 ± 0.88^b^
V + M	22.3 ± 0.88^b^	26.3 ± 0.57^b^	1.22 ± 0.05^b^	0.26 ± 0.07^b^	7.33 ± 0.05^b^

C = untreated control, M = colonized with AMF, and V = infected with ToMV.

*Values of each column followed by the same letter are not significantly different according to Duncan’s multiple range test (*P* ≤ 0.05), each value represents the mean of four replicates ± SD.

### Effects on the photosynthetic pigments and total flavonoid content

Means of the photosynthetic pigments and the total flavonoid content of tomato plants in response to the different applied treatments are presented in (Table 4). Infection of tomato plants with ToMV led to a significant reduction in the estimated photosynthetic pigments (Chl. *a* and *b*, and carotenoids) compared to the control plants. However, colonization of tomato roots with AMF significantly enhanced all of the estimated pigments recording the highest values in this concern compared with the control plants. Mycorrhizal colonization of the ToMV-infected plants significantly enhanced the photosynthetic pigments compared with the non-mycorrhizal-infected plants. On the other hand, all applied treatments led to significant increases in the total flavonoid content of the tomato plants compared with the control plants. Total flavonoid content of the ToMV-infected plants was higher than that of the AMF-colonized plants when compared with the control plants. However, the highest total flavonoid content was noticed for the ToMV-infected-AMF-colonized plants.

**Table 4:** Effect of root colonization with AMF on the photosynthetic pigments (mg g^−1^ fresh wt) and total flavonoid content (mg rutin equivalents 100 g^−1^ fresh wt) in the leaves of tomato plants infected with ToMV (21 days after inoculation).

Treatment*	Chl. *a*	Chl. *b*	Carotenoids	Total flavonoids
C	2.18 ± 0.27^b^	1.42 ± 0.16^b^	0.78 ± 0.05^b^	223 ± 4.32^d^
M	2.59 ± 0.32^a^	1.73 ± 0.14^a^	0.85 ± 0.04^a^	298 ± 5.66^c^
V	1.77 ± 0.15^c^	1.05 ± 0.12^c^	0.63 ± 0.06^c^	381 ± 3.45^b^
V + M	2.14 ± 0.22^b^	1.40 ± 0.11^b^	0.77 ± 0.05^b^	497 ± 6.27^a^

C = untreated control, M = colonized with AMF, and V = infected with ToMV.

*Values of each column followed by the same letter are not significantly different according to Duncan’s multiple range test (*P* ≤ 0.05), each value represents the mean of four replicates ± SD.

### Transcript levels of the polyphenol biosynthesis-related genes

**Figure 3:**
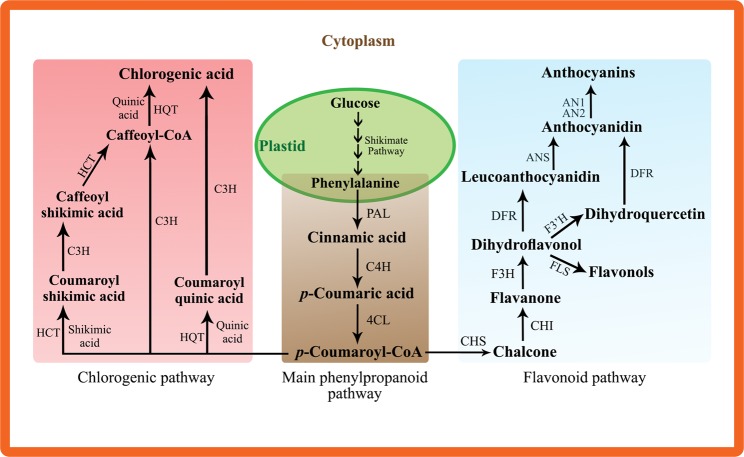
Graphical overview of the phenylpropanoid, flavonoid and chlorogenic acid biosynthetic pathways and their regulating genes (adapted from André *et al*.^34^; Mahesh *et al*.^53^; Albert *et al*.^54^). where, *PAL1*: phenylalanine ammonia-lyase1, *C4H*: cinnamic acid 4-hydroxylase, *4CL*: 4-coumarate-CoA ligase, *CHS*: chalcone synthase, *CHI2*: chalcone isomerase 2, *F3H*: flavanone 3-hydroxylase, *F3*′*H*: flavonoid 3′ hydroxylase, *FLS1*: flavonol synthase 1, *DFR*: dihydroflavonol 4-reductase, *ANS*: anthocyanidin synthase, *AN1*: anthocyanin 1 transcription factor, *AN2*: anthocyanin 2 transcription factor, *HQT*: hydroxycinnamoyl Co A quinate hydroxycinnamoyl transferase, *C3H*: *p*-coumarate 3-hydroxylase, and *HCT*: hydroxycinnamoyl Co A shikimate hydroxycinnamoyl transferase.

Expression levels of thirteen genes encoding the enzymes set catalyzing the polyphenol biosynthetic pathways were investigated 21 days after ToMV inoculation. The polyphenol biosynthetic pathway can be divided into three sections; the main phenylpropanoid biosynthetic pathway, the flavonoid biosynthetic pathway, and the chlorogenic acid biosynthetic pathway (Fig. 3).

### The main phenylpropanoid biosynthetic pathway

**Figure 4:**
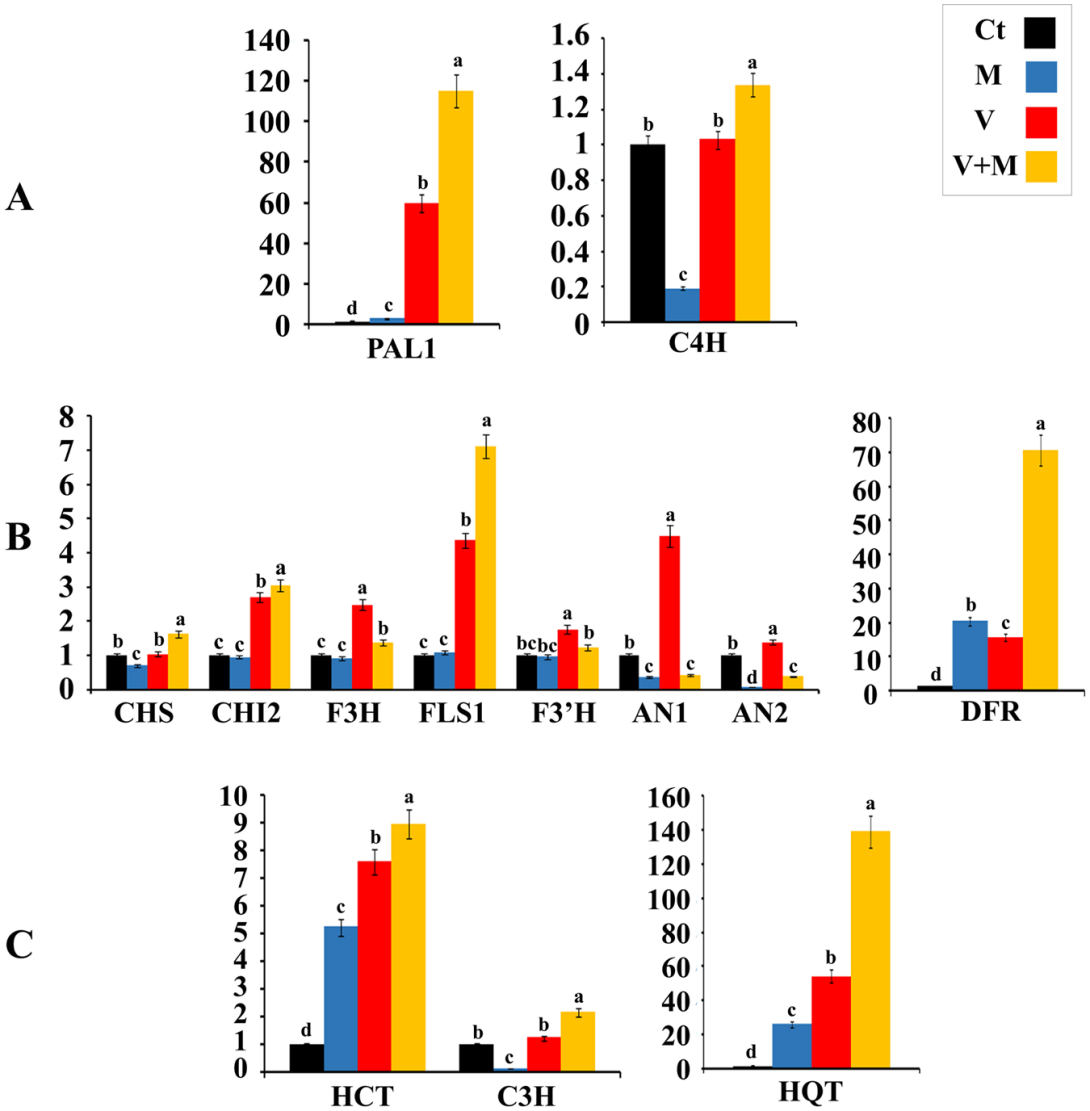
Histograms showing the relative transcriptional expression levels of phenylpropanoid (**A**), flavonoid (**B**) and chlorogenic acid (**C**) biosynthetic pathways genes in tomato as response to mycorrhizal colonization and infection with ToMV. Where, Ct = untreated control, M = colonized with AMF, V = infected with ToMV, and V + M = infected with ToMV and colonized with AMF. Columns superscripted with the same letter(s) are not significantly different according to Duncan’s multiple range test (*P* ≤ 0.05). Each value represents the mean of three biological replicates, each analyzed in triplicate. Error bars represent standard errors.

Transcript levels of *PAL1* and *C4H* genes, which control the first two steps in this pathway, were investigated. For *PAL1* expression, treatment with either ToMV or AMF triggered the gene expression level, but the gene up-regulation in the ToMV-infected plants was much higher (60-fold) than that in the AMF-colonized plants when compared with the control plants (Fig. 4A). However, the highest *PAL1* transcript level was observed for the ToMV-infected-AMF-colonized plants (116-fold). The expression level of *C4H* was not induced in the ToMV-infected plants, while, it decreased in the AMF-colonized plants compared with the control plants (Fig. 4A). Nevertheless, the expression level increased in the ToMV-infected-AMF-colonized plants (1.4-fold). Based on the obtained results, two points were noticed. The first, the relative expression level of *PAL1* was much higher than that of *C4H*, suggesting that both genes are not expressed in a coordinated manner. The second, the up-regulated expression of both genes by the dual treatment (ToMV + AMF) was higher than that of the single treatments, suggesting their synergistic effect on both genes, and the pathogen-dependent inducing effect of AMF.

### The flavonoid biosynthetic pathway

Transcriptional expression profiles of eight genes controlling the flavonoid biosynthesis pathway (*CHS*, *CHI2*, *F3H*, *FLS1*, *DFR*, *F3*′*H*, *AN1*, and *AN2*) are illustrated in Fig. (4B). The obtained results indicated that the expression level of *CHS* was not induced by ToMV infection, while AMF colonization down-regulated the gene expression compared with the control treatment. A significant expression of *CHS* was observed for the ToMV + AMF treatment recording 1.7-fold increase. In regard to *CHI2*, an increased expression was displayed for the ToMV infection, while the mycorrhizal colonization did not affect the gene expression when compared with the control plants. Treatment with ToMV + AMF showed the highest expression level of *CHI2* (3-fold). Concerning F3H gene, infection of tomato plants with ToMV singly or with AMF colonization up-regulated the gene expression, but the single treatment exhibited the highest expression level (2.5-fold) compared to the control plants, whilst, colonization of tomato roots with AMF did not affect the *F3H* expression level. Both treatments with ToMV, singly or with AMF, enhanced the transcript level of *FLS1* gene. The ToMV + AMF treatment showed the highest enhancing effect on the *FLS1* expression level (7.1-fold), compared to the control treatment. With regard to *DFR* gene, all treatments up-regulated the gene expression but the AMF treatment was more enhancer than the ToMV treatment when compared with the control treatment. The most highly expression level was noticed for the ToMV + AMF treatment, recording 71-fold increase. Viral infection with ToMV alone or in the presence of AMF increased the expression level of *F3*′*H* gene, but the single treatment (ToMV) was the most enhancing treatment, compared with the control treatment, while the mycorrhizal colonization had no effect on the transcript level of *F3*′*H*. Infection with ToMV up-regulated the expression level of *AN1* recording 4.5-fold increase, while colonization with AMF alone or in the infected plants down-regulated the gene expression. The same observations were noticed for the transcript level of *AN2* gene, where infection with ToMV up-regulated the *AN2* gene expression level as 1.4-fold than the control treatment, while the mycorrhizal plants showed significantly low transcript level, in the presence or absence of ToMV.

### The chlorogenic acid biosynthetic pathway

Expression pattern of three genes which regulate the chlorogenic acid biosynthesis pathway (*HQT*, *HCT*, and *C3H*) are illustrated in Fig. (4C). For *HQT*, the transcript level was increased by virus infection and mycorrhizal colonization, but the induction in ToMV-infected plants was higher than that in AMF-colonized plants when compared with the control plants. However, tomato plants treated with ToMV + AMF showed the highest up-regulation level (140-fold) compared with the other plants. The obtained results indicated that the expression level of *HCT* in tomato plants was induced by both viral and mycorrhizal treatments, but the viral induction was higher than the mycorrhizal one when compared with the control plants. However, treating of tomato plants with ToMV + AMF showed the highest transcript level of this gene (9-fold). On the other hand, the obtained data showed that the *C3H* gene was not induced by the ToMV infection, while the mycorrhizal colonization down-regulated it compared with the control treatment. However, treatment with ToMV + AMF exhibited a significant expression level (2.2-fold). Expression patterns of the three genes indicated that the transcriptional regulation of *HQT* was the highest compared to the other genes. In addition, the pathogen-dependent triggering effect of AMF was also noticed for the three genes regulating the chlorogenic acid pathway.

### Accumulation level of ToMV

**Figure 5:**
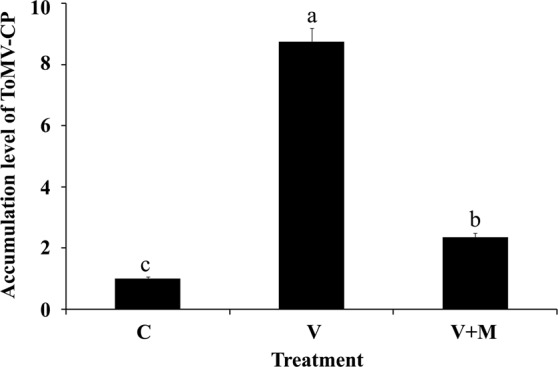
A histogram showing the relative transcriptional expression level of *ToMV-CP* gene in ToMV-infected tomato plants (21 days after ToMV inoculation) in response to mycorrhizal colonization with AMF. Where, C = untreated control, V = infected with ToMV, and V + M = infected with ToMV and colonized with AMF. Columns superscripted with the same letter(s) are not significantly different according to Duncan’s multiple range test (*P* ≤ 0.05). Each value represents the mean of three biological replicates, each analyzed in triplicate.

The obtained results exhibited a high accumulation level of ToMV (8.74-fold) in the ToMV- infected plants. A considerable reduction in the viral accumulation level in tomato plants treated with ToMV + AMF (2.36-fold) was observed when compared with the control plants (Fig. 5).

## Discussion

Biocontrol activity of AMF has been extensively studied against various plant diseases^12,14,15,22^. Nevertheless, reports dealing with the biocontrol activity of AMF against viral plant diseases are limited. This study deals with the biocontrol activity of AMF against ToMV on tomato plants, particularly their effects on the transcriptional regulation of flavonoid and chlorogenic acid biosynthetic pathways genes.

One of the widely reported beneficial effects of mycorrhizal association is enhancing of the host plant growth^23^. In this regard, data obtained in this study showed the enhancing effect of the mycorrhizal colonization on the evaluated tomato growth parameters as well as the photosynthetic pigments content. This result is in agreement with that reported by Lin *et al*.^24^ on *Leymus chinensis* plants. Mycorrhizal association improves the water and nutrients uptake of the host plant through the external mycelial network of AMF in the rhizospheric soil^25^. Production of growth hormones in mycorrhizal plants has also been described. These endogenous hormones improve the nutrients translocations, plant photosynthetic activity and metabolism^26^. This may explain the enhancing effect of AMF on the growth of tomato plants in this study. On the other hand, it is recognized that the vigorous plant is more able to resist the invading pathogens than the weak one. Moreover, plant growth enhancement by AMF plays an important role in the compensation for the disease damages.

Results obtained from the greenhouse experiment confirmed the effective biocontrol activity of root mycorrhizal colonization against infection of tomato plants with ToMV, which resulted in a considerable reduction in the viral accumulation level. This result is in line with that of Maffei *et al*.^16^ who reported a remarkable attenuation in the disease symptoms of tomato plants infected with tomato yellow leaf curl Sardinia virus, as well as the concentration of the viral DNA, when colonized with AMF. Induction of systemic acquired resistance (SAR) in the host plant due to mycorrhizal colonization has been described by many researchers^13,27^. This resistance is mediated by jasmonate-dependant signaling pathway^28^. A number of hypersensitivity responses including physical, biochemical and molecular defense-related changes has been reported to be associated with induction of SAR such as cell wall lignification, accumulation of pathogen-toxic substances like phenolics, enzymes, and/or pathogenesis-related proteins^13^. Furthermore, up-regulation of some defense-related genes has also been reported^29^. Among the phenolic compounds induced by SAR, this study focused on the flavonoids and chlorogenic acid. Results obtained in this study revealed a pathogen-dependent inducing effect by AMF on the transcriptional levels of the majority of the studied genes. Moreover, this induction was coordinated with the highest flavonoid content in ToMV-infected-AMF-colonized plants, compared with the other treatments. This result is in agreement with that obtained by Marquez *et al*.^30^ who reported major transcriptional up-regulations for the defense-related genes encoding simple phenols, flavonoids, and lignin in soybean plantlets infected with *Fusarium virguliforme* and colonized with AMF. Interestingly, the mycorrhizal + infected soybean plantlets demonstrated the largest number of up-regulated genes. Flavonoids and chlorogenic acid have various functions in the plant including resistance against biotic and abiotic stresses. Their antiviral activity has been extensively reported against various plant and human viruses^19,20^. In this regard, Krcatovic *et al*.^31^ reported the antiviral activity of the two flavonoids, quercetin and vitexin, against *Tobacco Mosaic Virus* (TMV) on tobacco plants, where, the viral concentration was significantly reduced in the infected plants. Seven flavonoids, isolated from *Cassia fistula*, demonstrated varying degrees of antiviral potency against TMV, recording inhibition rates ranged between 18.5 and 31.3%, compared to 24.7% for ningnanmycin^32^. Furthermore, chlorogenic acid also showed antiviral activity against influenza A (H1N1/H3N2) virus^33^. The antiviral mechanisms utilized by flavonoids and chlorogenic acid revolve around their enzyme-inhibitory effects, particularly, against viral polymerase and integrase, in addition to their binding ability to the viral nucleic acid and/or capsid protein^21^. One of the most important observations in this study is the most highly transcriptional expression levels of *PAL1* and *HQT* compared to the other up-regulated genes. Indeed, *HQT* is the primary route for the chlorogenic acid biosynthesis in the solanaceous plants. *HQT* catalyzes the conversion of caffeoyl CoA to chlorogenic acid, as well as the reaction which converts *p*-coumaroyl CoA to coumaroyl quinic acid, which will be then converted into chlorogenic acid (Fig. 3)^34^. Zhang *et al*.^35^ reported a positive correlation between the chlorogenic acid content and the transcriptional level of HQT in* Lonicera japonica*. Moreover, *HQT*-silenced tomato plants showed 98% reduction in the chlorogenic acid content than the wild-type plants^36^. On the other hand, *PAL1*-encoding gene regulates the first step in the main phenylpropanoid pathway which represents the start point for the biosynthesis of many important substances such as flavonoids, coumarins, and lignans^37^. *PAL1* catalyzes the conversion of phenylalanine to *trans*-cinnamic acid, the phenylpropanoid skeleton, which is then used in the biosynthesis of flavonoids (Fig. 3)^38^.

The *C4H* encoding gene, which regulates the hydroxylation of *t*-cinnamic acid to form *p*-coumaric acid, the second step in the main phenylpropanoid pathway, was also up-regulated by AMF in the presence of ToMV. *HCT*, *C4H* and *C3H* have a defense-related function represented in their role in the biosynthesis of the monolignols used in the cell walls lignifications^39^. *HCT* catalyzes the biosynthsis of two major lignin building units (guaiacyl and syringyl)^40^. *HCT*-silenced *Nicotiana* plants exhibited considerable changes in the amount and composition of lignin and affects phenylpropanoid metabolism^41^. Induction of transcriptional expression of these genes indicates their protective role against ToMV. The *CHS* and *CHI2* encoding genes have also a protective role in the plant immunity through regulating the pathogen-dependant accumulation of flavonoids and isoflavonoid phytoalexins. Both enzymes catalyze the conversion of *p*-coumaroyl CoA into naringenin^42,43^. Up-regulation of *CHS* and *CHI2* encoding genes by mycorrhizal colonization in the ToMV-infected plants enhances the plant immunity against this virus. Likewise, transcriptional regulation of *FLS1*, *F3H* and *DFR* encoding genes leads to accumulation of flavonols supporting plant resistance against the viral accumulation, while, *F3*′*H* is involved in the flavonols-anthocyanins biosynthesis^38^. On the contrary, the transcriptional expression levels of *AN1* and *AN2* encoding genes, which regulate anthocyanins biosynthesis, were down-regulated by AMF colonization. In conclusion, the present study showed the growth enhancing effect of mycorrhizal colonization with AMF on tomato plants and confirmed their role in triggering plant immunity against ToMV. Transcriptional up-regulation of most of the studied genes by AMF in a pathogen-dependent manner, particularly *PAL1* and *HQT*, may elucidate their protective role against ToMV.

## Materials and Methods

### Viral inoculum and tomato cultivar

Tomato plant infected with ToMV was used to prepare the viral inoculum. Infected young leaves were grinded to collect the crude cell sap using 0.1 M phosphate buffer (pH 7). The ToMV-susceptible tomato plant (cv. Ailsa Craig) was used in this study.

### AMF inoculum

A mixed AMF inoculum propagated under sudangrass (80% colonization index) was used in this study. The used AMF were *Funneliformis mosseae* (T.H. Nicolson & Gerd.) C. Walker & A. Schüßler, *Rhizoglomus clarum* (T.H. Nicolson & N.C. Schenck) Sieverd., G.A. Silva & Oehl, and *Rhizophagus aggregatus* (N.C. Schenck & G.S. Sm.) C. Walker, in equal proportions. The AMF inoculum composed of rhizospheric soil with external mycelia and colonized root fragments from the pot culture.

### Evaluation of AMF application under greenhouse conditions

Plastic pots (20 cm-diameter) filled with sterilized soil (clay:sand, 1:1, v/v) were used. In each pot, five healthy 28-day-old tomato seedlings were transplanted. At transplanting time, half of the used pots were inoculated with AMF inoculum (50 g pot^−1^) as a seedling bed. No fertilization was applied and the pots were regularly irrigated with tap water to near filed capacity. Three weeks after transplanting, tomato plants were mechanically inoculated with a freshly prepared ToMV inoculum. For inoculation, tomato leaves were dusted with carborundum powder (600 meshes), then the ToMV inoculum was gently rubbed onto the dusted leaves using forefinger. Inoculated plants were rinsed with tap water shortly after the inoculation. Tomato plants only treated with plain sterilized water were used as a negative control. For each treatment, five pots were used as replicates. The pots were arranged in a complete randomized design. The tested treatments in this experiment were designated as follow; control (C), virus-infected (V), colonized with AMF (M), and virus-infected and colonized with AMF (V + M). All pots were kept under greenhouse conditions at 26/20 °C day/night and 65% relative humidity.

### Disease assessment

Twenty one days after ToMV inoculation, the disease symptoms were observed in all infected pots as described by Mansour and Al-Musa^44^. Disease severity of the infected plants was scored on a six-degrees-scale based on the disease symptoms and leaf damage according to Imran *et al*.^45^ as follows: 0 = no symptoms, 1 = 1–20%, 2 = 21–40%, 3 = 41–60%, 4 = 61–80%, and 5 = 81–100%. Values of the disease severity were then transformed to percent disease index (PDI) using the following formula:

$${\rm{PDI}}=\frac{{\sum }^{}ab}{AK}\times 100$$

where *a* = number of diseased plants having the same severity grade, *b* = severity grade, *A* = total number of plants and *K* = highest degree of infection. The disease incidence was calculated using the following formula:

$${\rm{Disease}}\,{\rm{incidence}}\,( \% )=\frac{{\rm{Number}}\,{\rm{of}}\,{\rm{infected}}\,{\rm{plants}}}{{\rm{Total}}\,{\rm{number}}\,{\rm{of}}\,\mathrm{plants}\,}\times {\rm{100}}$$

where 1–20% = low incidence, 21–49% = moderate incidence, and 50–100% = high incidence.

### Growth parameters evaluation

Four random plants from each treatment were carefully uprooted, washed under running water, and assessed for the shoot and root lengths (cm), shoot and root dry weight (g), and number of leaves. Dry weights were determined after drying the plant samples in an oven at 80 °C for 72 h.

### Estimation of mycorrhizal colonization in tomato roots

Four tomato plants from each treatment were carefully uprooted (with their entire roots) and washed under running water to remove soil particles. The roots were cut into small segments (1 cm), and stained with 0.05% trypan blue (Sigma, St. Louis, MO) according to Phillips and Hayman^46^. For each treatment, forty root segments were mounted on slides in lactoglycerol and examined microscopically for estimation of mycorrhizal root colonization according to Trouvelot *et al*.^47^. Frequency of root colonization (F, %), intensity of cortical colonization (MI, %), and frequency of arbuscules (A, %) were estimated.

### Biochemical analysis

The photosynthetic pigments (chlorophyll *a*, chlorophyll *b*, and carotenoids) were estimated in leaves of the treated tomato plants according to Harborne^48^. Extraction and estimation of the total flavonoid content were preceded according to Jia *et al*.^49^.

### Expression analysis of polyphenol-pathway genes

#### Total RNA extraction and cDNA synthesis

Total RNA was extracted from the treated tomato leaves using RNeasy Mini Kit (Qiagen, Germany) according to the manufacturer’s instructions and dissolved in diethyl pyrocarbonate-treated water. The extracted RNA was then incubated with DNase for 1 h at 37 °C and quantified using a NanoDrop 1000 spectrophotometer (Thermo Scientific, USA).

Reverse transcription reaction was performed in a reaction mixture (20 µL) containing 2.5 µL 10×-buffer with MgCl_2_, 2.5 µL of dNTPs (10 mM), 1 µL oligo (dT) primer (10 pmol µL^−1^), 3 µL RNA (30 ng) and 0.2 µL reverse transcriptase enzyme (M-MuLV Reverse Transcriptase, Biolabs, NewEngland) and 10.8 µL sterile water. The PCR was performed using a SureCycler 8800 thermocycler (Agilent Technologies, USA) at 42 °C for 2 h, then at 70 °C for 5 min and the cDNA was then stored at −20 °C until used.

#### Quantitative Real-Time PCR (qRT-PCR)

The qRT-PCR reaction consisted of 10 μL 2xSYBR^®^ Green RT Mix (Bioloine, Germany), 1 µL of each forward and reverse primers (10 pmol µL^−1^), 1 µL cDNA (50 ng) and up to 7 µL of RNase free water. Sequences of the primers used in this study are presented in (Table 5). The real time PCR program was performed using a Rotor-Gene-6000-system (Qiagene, USA) as follows: one cycle at 95 °C for 15 min, 45 cycles (95 °C for 15 sec, 60 °C for 30 sec and 72 °C for 30 sec). A *β*-actine gene was used as a reference gene (forward 5′-GTGGGCCGCTCTAGGCACCAA-3′and reverse 5′-CTCTTTGATGTCACGCACGATTTC-3′). For each sample, three biological and three technical replicates were performed. The comparative C_T_ method (2^−ΔΔCT^) was used to analyze the relative mRNA expression levels of the tested genes^50^.

**Table 5:** Primer sequences of flavonoid and chlorogenic acid biosynthetic pathways genes.

Primer	Solyc ID	Abver	Sequence (5′-3′)
Phenylalanine ammonia-lyase 1	Solyc10g086180.1	PALF PALR	ACGGGTTGCCATCTAATCTGACA CGAGCAATAAGAAGCCATCGCAAT
Cinnamic acid 4-hydroxylase	Solyc05g047530.2	C4HF C4HR	CCC AGT TTT TGG AAA TTG GCT TCA GCC CCA TTC TAA GCA AGA GAA CAT C
Chalcone synthase	Solyc05g053550.2	CHSF CHSR	CAC CGT GGA GGA GTA TCG TAA GGC TGA TCA ACA CAG TTG GAA GGC G
Chalcone isomerase 2	Solyc02g062170	CHIF CHIR	GGC AGG CCA TTG AAA AGT TCC CTA ATC GTC AAT GAT CCA AGC GG
Flavanone 3-hydroxylase	Solyc02g083860.2	F3HF F3HR	CCA AGG CAT GTG TGG ATA TGG ACC CCT GGA TCA GTA TGT CGT TCA GCC
Flavonol synthase 1	Solyc11g013110.1	FLSF FLSR	CCT CCT TCC TAC AGG GAA GCA AA CAA GCC CAA GTG ACA AGC TCC TAA
Dihydroflavonol 4-reductase	Solyc02g085020	DFRF DFRR	TCA CAG GAG CAG CTG GAT TTA TCG TCA GGA TCA CGA ACA GTA GCA TGG
Flavonoid 3′ hydroxylase	Solyc11g066580	F3′HF F3′HR	TGG GTA TAC CCA AAC TCA TTC CG AAA AGC CCA AAG TTG ATG TGA AAG G
Anthocyanin 1 transcription factor	Solyc10g086260	AN1F AN1R	CCT CAA CCT CAG AAA TTC AGA AGC TCG TTG TTGTTG TCG TTC GAT GC
Anthocyanin 2 transcription factor	Solyc10g086250	AN2F AN2R	ACAAGATGCCACTTTCCTTCACC TGTGCATCGTTGGGAGTTAGG
Hydroxycinnamoyl Co A shikimate hydroxycinnamoyl transferase	Solyc06g074710	HCTF HCTR	TCT CCA ACC CCT TTT AAC GAA CC CAA CTT GTC CTT CTA CCA CAG GGA A
Hydroxycinnamoyl Co A quinate hydroxycinnamoyl transferase	Solycg07g005760	HQTF HQTR	CCC AAT GGC TGG AAG ATT AGC TA CAT GAA TCA CTT TCA GCC TCA ACA A
*p*-coumarate 3-hydroxylase	Solyc10g078240	C3HF C3HR	TTG GTG GCT ACG ACA TTC CTA AGG GGT CTG AAC TCC AAT GGG TTA TTC C

#### Accumulation level of ToMV-CP using qRT-PCR

The qRT-PCR reaction consisted of 10 μl of 2x Quantitech SYBR® Green RT Mix (Bioline, Germany), 1 µL of each forward and reverse primers (10 pmol µL^−1^), 1 µL cDNA (50 ng), and up to 7 µL of RNase free water. The ToMV-specific primers covering coat protein (CP) gene was used (forward 5′-CGGAAGGCCTAAACCAAAAAG-3′, reverse 5′-ATTTAAGTGGAGGGAAAAACACT-3′) according to Letschert *et al*.^51^. The real time PCR program was performed using a Rotor-Gene-6000-system (QIAGEN, USA) as follows: one cycle at 95 °C for 5 min, 45 cycles (95 °C for 15 sec, 60 °C for 30 sec and 72 °C for 30 sec). *18S rRNA-F* (5′-TACCTGGTTGATCCTGCCAGTAG-3′) and *18S rRNA-R* (5′-CCAATCCCTAGTCTGCATCGT-3′) were used as a reference gene. The qRT-PCR reactions were done in three biological replicates, each was analyzed in triplicate as the same as described above.

### Statistical analysis

All data were statistically analyzed using the software CoStat (version 6.4). Comparisons between the means were performed using Duncan’s multiple range test at *P* ≤ 0.05^52^.

## Supplementary information

Fig. X
