# Supplementary material for: Arbuscular Mycorrhizal Fungi Trigger Transcriptional Expression of Flavonoid and Chlorogenic Acid Biosynthetic Pathways Genes in Tomato against Tomato Mosaic Virus
Source: Sci Rep. 2019 Jul 4;9:9692. doi: 10.1038/s41598-019-46281-x (PMC6609724; doi:10.1038/s41598-019-46281-x)
Supplement: Supplementary file 1 — Fig. X [file 41598_2019_46281_MOESM1_ESM.pdf]

Arbuscular Mycorrhizal Fungi Trigger Transcriptional Expression of Flavonoid and Chlorogenic Acid Biosynthetic Pathways Genes in Tomato against *Tomato Mosaic Virus*

Dalia G. Aseel, Younes M. Rashad and Saad M. Hammad

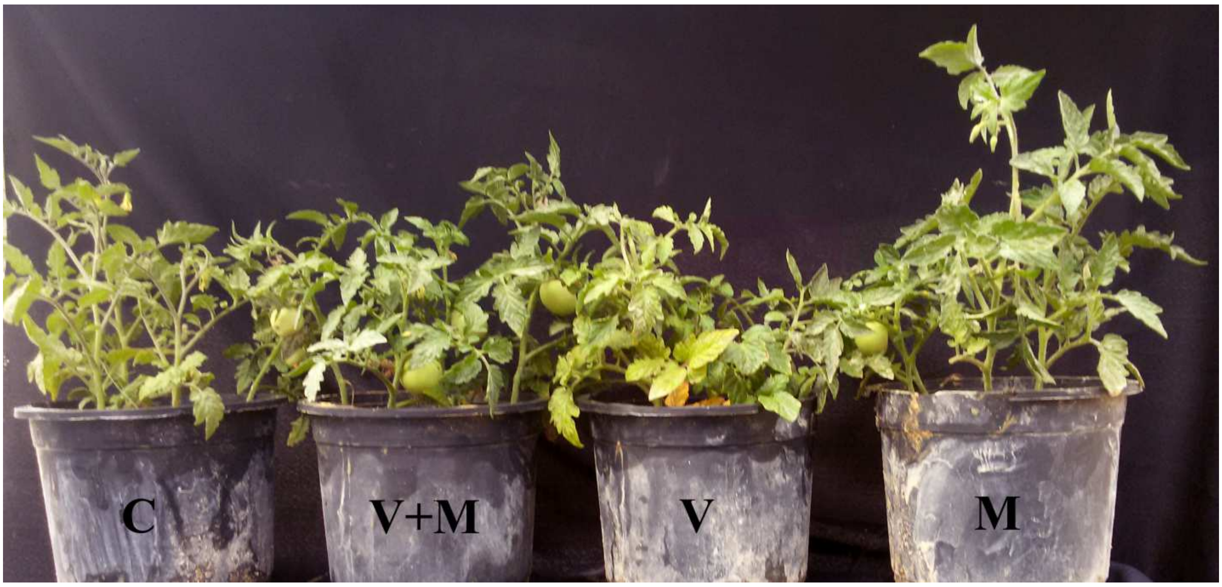

Fig . X: A photograph showing the whole plants of the applied treatments in the greenhouse experiment. Where, C = untreated control, M = colonized with AMF, V = infected with ToMV, and V+M = infected with ToMV and colonized with AMF.
